# Pre-Exercise Caffeine as a Modulator of Exercise-Induced Acute Cardiometabolic Responses: A Narrative Review

**DOI:** 10.3390/metabo16070478

**Published:** 2026-07-08

**Authors:** Haifeng Geng, Zhibo Zhou, Lingfei Meng, Mingnan Zhuang, Yan Zhao

**Affiliations:** 1 Department of Public Basic Courses, Chongqing Three Gorges Medical College, Chongqing 404120, China; genghaifeng199408@163.com; 2 Department of Environmental and Materials Engineering, Yantai Gold College, Yantai 264000, China; 3 School of Athletic Performance, Shanghai University of Sport, Shanghai 200438, China; zhouzhibo1993@163.com; 4 School of Physical Education, Chongqing University of Posts and Telecommunications, Chongqing 400065, China; menglingfei1994@163.com; 5 School of Sports Training, Tianjin University of Sport, Tianjin 301617, China; iammnz@163.com

**Keywords:** caffeine, exercise, cardiometabolic responses, hemodynamics, vascular function, substrate metabolism

## Abstract

Caffeine is among the most widely used supplements in sports nutrition, with substantial evidence supporting its efficacy for enhancing athletic performance. However, the relevance of pre-exercise caffeine supplementation extends beyond its ergogenic effects. Exercise itself acts as an acute cardiometabolic stressor, eliciting dynamic responses in blood pressure, heart rate, vascular tone, substrate utilization, and glucose regulation. Caffeine may further modify the magnitude, temporal profile, and recovery kinetics of these responses. This focused narrative review examines the acute cardiometabolic responses associated with pre-exercise caffeine supplementation and its potential regulatory effects. It outlines the underlying biological mechanisms, including adenosine receptor antagonism, hemodynamic and vascular pathways, and metabolic and substrate-utilization pathways, while synthesizing current evidence from the literature. Available evidence indicates that the acute effects of caffeine are highly context dependent. In some exercise settings, caffeine may promote lipolysis and fat oxidation; in others, particularly among susceptible individuals or under specific exercise conditions, it may be associated with greater acute cardiovascular or autonomic load, reflected by higher peripheral vascular resistance and blood pressure, altered vascular reactivity, or delayed post-exercise autonomic recovery. Its effects may vary according to dose, timing, supplement form, exercise modality and intensity, training status, habitual caffeine intake, genotype, sex, and hormonal status. Overall, pre-exercise caffeine should not be regarded solely as a uniformly beneficial ergogenic aid, but rather as a physiological modulator that may reshape the acute cardiometabolic milieu during and after exercise. Future studies should integrate hemodynamic, vascular, metabolic, and individual-variability measures over extended observation periods to support more evidence-based and individualized guidance on the appropriate use of pre-exercise caffeine.

## 1. Introduction

Caffeine is a naturally occurring methylxanthine alkaloid found in plant sources such as coffee, tea, cocoa, and guarana, and is among the most widely consumed psychoactive substances worldwide [1,2,3]. Owing to its stimulatory effects on the central nervous system, caffeine can increase alertness, enhance attention-related functions, and reduce perceived fatigue. Substantial evidence also indicates that caffeine produces consistent ergogenic effects across a range of athletic tasks, thereby improving physical capacity and exercise performance [4,5,6]. Accordingly, caffeine has become one of the most widely used supplementation strategies in sports nutrition, with pre-exercise ingestion representing its most common practical application [5].

However, the relevance of pre-exercise caffeine supplementation should not be limited to its performance-enhancing effects. Exercise itself acts as an acute cardiometabolic stressor, eliciting dynamic physiological responses during both exercise and recovery, including changes in heart rate, blood pressure, blood flow redistribution, vascular tone, substrate mobilization, glucose homeostasis, and post-exercise recovery processes [7,8,9,10]. Whether pre-exercise caffeine ingestion further alters the magnitude, temporal profile, and recovery kinetics of these acute cardiometabolic responses therefore warrants careful consideration. Mechanistically, there is a clear biological rationale for caffeine to influence these processes. As a non-selective adenosine receptor antagonist, caffeine can modulate autonomic nervous system activity, influence circulating catecholamine concentrations, alter peripheral vascular resistance, and affect substrate mobilization and energy metabolism, thereby potentially shaping hemodynamic, vascular, and metabolic responses during exercise [11,12,13]. Nevertheless, research on caffeine and exercise has predominantly focused on its ergogenic properties, particularly its capacity to enhance endurance performance, high-intensity exercise capacity, and sport-specific performance [4,14,15,16,17]. By contrast, evidence on whether caffeine modulates acute exercise-induced cardiometabolic responses remains insufficiently integrated in a dedicated review.

Importantly, the effects of caffeine on acute cardiometabolic responses to exercise are unlikely to be unidirectional. Available evidence suggests that pre-exercise caffeine intake may, under certain conditions, promote lipolysis, enhance fat oxidation, and improve specific metabolic responses [18,19,20]. In other contexts, however, it may transiently elevate blood pressure, alter vascular reactivity, and impose additional cardiovascular strain in susceptible populations [21,22]. Thus, simply characterizing caffeine as either “beneficial” or “harmful” fails to capture the complexity of the evidence. A more nuanced perspective recognizes that caffeine’s effects on acute exercise-induced cardiometabolic responses are highly context dependent, with both their direction and magnitude shaped by multiple factors, including dose, timing of intake, supplement form, exercise modality, and individual characteristics [5,12]. Moreover, current evidence remains fragmented across sources, intervention modalities, and methodological designs. Relevant studies are dispersed across sports nutrition, exercise physiology, cardiovascular medicine, and metabolic research, and vary considerably in how caffeine exposure is defined. Some studies examine anhydrous caffeine, whereas others use coffee, energy drinks, or multi-ingredient pre-workout supplements. Because these delivery forms may contain physiologically active compounds in addition to caffeine, attributing observed effects specifically to caffeine itself and directly comparing findings across studies are inherently challenging [23]. Substantial heterogeneity also exists in dose protocols, exercise regimens, participant characteristics, and outcome measures, preventing the development of a unified conceptual framework for this topic.

In light of these considerations, a dedicated review is needed to examine the intersection between pre-exercise caffeine supplementation and acute exercise-induced cardiometabolic responses. This focused narrative review synthesizes evidence on pre-exercise caffeine supplementation as a modulator of acute exercise-induced cardiometabolic responses by examining its biological underpinnings, preclinical and human evidence, key influencing factors, practical implications, and remaining research gaps. Ultimately, this review aims to provide a clearer theoretical basis for understanding the physiological significance and practical application of pre-exercise caffeine supplementation.

## 2. Literature Identification and Selection

This article was designed as a focused narrative review rather than a systematic review or meta-analysis; therefore, no formal protocol registration, risk-of-bias assessment, or quantitative evidence synthesis was performed. Nevertheless, to enhance transparency, the literature was identified through a structured and iterative search strategy. Searches were conducted in PubMed/MEDLINE, Web of Science, and Scopus from database inception to 20 April 2026. Additional relevant articles were identified by screening the reference lists of key reviews, position stands, consensus statements, and eligible primary studies.

The search strategy combined terms related to caffeine exposure, exercise, and acute cardiometabolic outcomes. Search terms included combinations of “caffeine”, “coffee”, “energy drink”, “pre-workout supplement”, “exercise”, “physical activity”, “endurance exercise”, “resistance exercise”, “high-intensity interval training”, “blood pressure”, “heart rate”, “hemodynamic”, “vascular function”, “endothelial function”, “flow-mediated dilation”, “arterial stiffness”, “pulse wave velocity”, “fat oxidation”, “substrate metabolism”, “glucose”, “insulin”, and “cardiometabolic response”. Search terms were adapted as appropriate for each database.

Articles were considered eligible if they: (1) examined caffeine, coffee, energy drinks, or caffeine-containing supplements administered before exercise or in a context directly relevant to exercise; (2) reported acute cardiometabolic outcomes, including hemodynamic responses, vascular function, substrate utilization, glucose or insulin regulation, autonomic recovery, or related physiological mechanisms; (3) provided human experimental evidence, controlled trial data, randomized crossover findings, or mechanistic evidence relevant to the biological pathways discussed in this review; and (4) were full-text, peer-reviewed articles published in English. When interpreting acute cardiometabolic responses during or after exercise, human studies were prioritized. Preclinical, in vitro, and other mechanistic studies were used only to support biological plausibility, particularly where direct human exercise evidence was limited or unavailable.

Articles were excluded if they: (1) focused solely on chronic caffeine or coffee consumption without an acute pre-exercise component; (2) examined exercise performance outcomes without reporting cardiometabolic, vascular, metabolic, or recovery-related variables; (3) involved multi-ingredient supplements in which the contribution of caffeine could not be reasonably interpreted; (4) were conference abstracts, editorials, commentaries, or non-peer-reviewed reports; or (5) were not directly relevant to the interaction between caffeine, exercise, and acute cardiometabolic regulation. Review articles, meta-analyses, and position stands were used to contextualize the evidence base but were not treated as primary evidence for specific physiological outcomes.

Given the narrative scope of this review, the aim was not to provide an exhaustive enumeration of all available studies, but to synthesize representative and mechanistically informative evidence across hemodynamic, vascular, metabolic, and individual-variability domains. This approach allowed integration of direct human evidence with mechanistic findings while maintaining a clear distinction between empirical observations and biological interpretation.

## 3. Biological Basis for a Caffeine–Exercise Interaction

### 3.1. Adenosine Receptor Antagonism as the Initiating Mechanism

A key mechanism underlying the interaction between caffeine and exercise is caffeine’s pharmacological action as a competitive adenosine receptor antagonist. Structurally, caffeine resembles adenosine, an endogenous purine nucleoside dynamically generated through ATP hydrolysis that exerts broad neuromodulatory effects in both the central and peripheral nervous systems [24]. Four subtypes of G protein-coupled adenosine receptors have been identified to date—A1, A2A, A2B, and A3—each with distinct tissue distributions and signaling properties [25,26]. Among these, A1 and A2A receptors are considered the principal targets mediating caffeine’s physiological effects. Importantly, substantial inter-individual variation exists in adenosine receptor density and signaling sensitivity, and chronic or repeated caffeine exposure can induce adaptive regulation of receptor expression [27].

**Figure 1:**
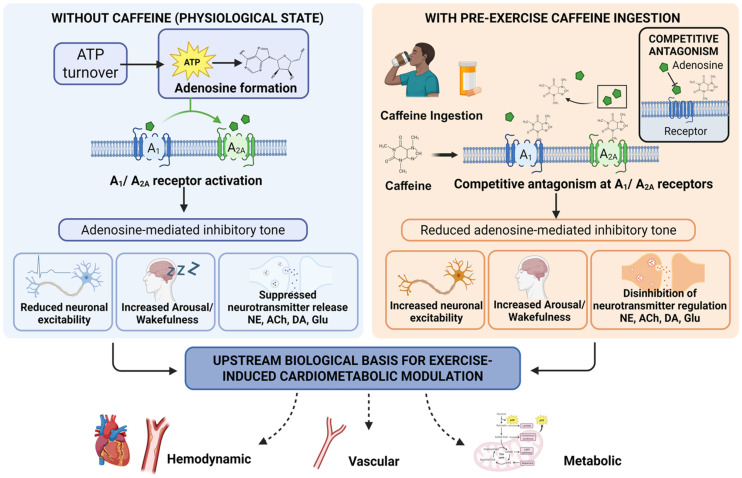
Adenosine receptor antagonism by caffeine as a proposed upstream mechanistic pathway linking caffeine intake to acute cardiometabolic responses during exercise. Caffeine (1,3,7-trimethylxanthine) is structurally analogous to adenosine and acts as a competitive antagonist at G-protein-coupled adenosine receptors. At plasma concentrations achievable with low-to-moderate dietary intake (low micromolar range), caffeine’s actions are mediated predominantly through blockade of the A1 and A2A subtypes, whereas phosphodiesterase inhibition and direct sarcoplasmic Ca^2+^ release require supraphysiological concentrations and are not considered primary mechanisms in this dose range. Under basal conditions, tonic activation of A1/A2A receptors by endogenous adenosine constrains presynaptic release of norepinephrine (NE), acetylcholine (ACh), dopamine (DA), and glutamate (Glu), and limits neuronal excitability. Caffeine-induced disinhibition may lift this inhibitory tone and increase central arousal and sympathoadrenal output, thereby providing a plausible neurobiological background for downstream hemodynamic, vascular, and metabolic responses to exercise. A1, adenosine A1 receptor; A2A, adenosine A2A receptor; A2B, adenosine A2B receptor; A3, adenosine A3 receptor; ACh, acetylcholine; ATP, adenosine triphosphate; DA, dopamine; Glu, glutamate; NE, norepinephrine; PDE, phosphodiesterase.

Within the range of plasma concentrations relevant to sports nutrition practice, typically micromolar levels achieved after low-to-moderate caffeine intake, caffeine’s primary mechanism of action is unlikely to involve phosphodiesterase (PDE) inhibition or direct intracellular Ca^2+^ release. These effects generally become physiologically relevant only at concentrations that substantially exceed those achieved through typical dietary or supplemental intake. Instead, competitive blockade of A1 and A2A adenosine receptors is widely regarded as the central mechanism underlying caffeine’s physiological effects during both habitual intake and exercise (Figure 1) [28,29,30,31]. Under basal physiological conditions, adenosine maintains a persistent inhibitory tone by activating these receptors, thereby suppressing neuronal excitability and limiting the release of several neurotransmitters. By blocking this pathway, caffeine reduces adenosine-mediated suppression of the central nervous system, increases arousal and neuronal firing, and provides an important neurobiological basis for subsequent cardiovascular and metabolic regulatory responses during exercise stress.

Preclinical evidence further supports this mechanism. Adenosine, particularly through A1 receptor activation, inhibits the release of neurotransmitters involved in central excitability and neural network regulation, including norepinephrine [32], acetylcholine [33,34], dopamine [35], and glutamate [36]. Adenosine receptor antagonists such as caffeine can partially relieve this inhibition. In addition, functional interactions between A2A receptors and dopaminergic signaling pathways suggest that caffeine’s central stimulant effects likely arise from the broader disinhibition of a multi-neurotransmitter network, driven by concurrent antagonism of A1 and A2A receptors, rather than from blockade of a single receptor pathway alone [37,38]. For the acute cardiometabolic responses to exercise considered in this review, adenosine receptor antagonism should not be interpreted as direct evidence for any specific cardiovascular or metabolic outcome. Rather, it provides an upstream mechanistic framework through which caffeine may influence downstream physiological domains relevant to exercise, including hemodynamic regulation, vascular reactivity, and substrate utilization. The mechanistic basis for these links is discussed in detail in the following sections on hemodynamic–vascular and metabolic pathways, whereas direct human evidence for these outcomes is synthesized separately in Section 4.

### 3.2. Hemodynamic and Vascular Pathways

Exercise-induced hemodynamic adjustments are a central component of acute cardiometabolic stress, involving progressive increases in cardiac output, redistribution of peripheral blood flow, and heightened perfusion demands in working skeletal muscle. Pre-exercise caffeine intake may modulate these processes through multiple interconnected pathways, thereby altering circulatory regulation during exercise and recovery.

From the perspective of systemic circulation, adenosine is an important mediator of metabolic–vascular coupling. Under conditions of local hypoxia, metabolite accumulation, and increased tissue perfusion demand, adenosine contributes to functional hyperemia and reactive vasodilation, primarily through activation of adenosine receptors on vascular smooth muscle and endothelial cells, thereby helping maintain the balance between local oxygen supply and demand [39,40]. By competitively antagonizing A1 and A2A receptors, caffeine may attenuate this endogenous vasodilatory signaling, increase total peripheral resistance, and provide a mechanistic basis for acute elevations in blood pressure and cardiac afterload [41]. This effect is particularly relevant during exercise, when perfusion is regulated by an intricate interaction among metabolite accumulation, including adenosine, lactate, H^+^, and K^+^; increased shear stress; sympathetic vasoconstrictor drive; and local vasodilatory signals. By interfering with adenosine-mediated vasodilation, caffeine may shift the balance between local perfusion of working muscles and systemic blood pressure maintenance, thereby influencing blood pressure trajectories, blood flow distribution, and local perfusion efficiency during exercise.

However, attributing caffeine’s vascular effects solely to a pro-constrictive action oversimplifies its multifaceted influence. At the endothelial level, caffeine can modulate intracellular Ca^2+^ dynamics and promote Ca^2+^/calmodulin-dependent activation of endothelial nitric oxide synthase (eNOS), thereby increasing nitric oxide (NO) production [41]. NO subsequently diffuses into adjacent vascular smooth muscle cells and induces vasodilation through the soluble guanylate cyclase (sGC)–cyclic guanosine monophosphate (cGMP) signaling pathway [41,42]. In parallel, the endothelium can further regulate vascular tone through the release of other vasodilatory mediators, including prostacyclin and endothelium-derived hyperpolarizing factor (EDHF) [43,44,45,46]. At the vascular smooth muscle level, caffeine may also influence cyclic nucleotide signaling and Ca^2+^ handling. Under certain conditions, partial inhibition of phosphodiesterase (PDE) may delay cAMP and cGMP degradation, reduce myosin light-chain kinase (MLCK) activity, and attenuate IP_3_-mediated sarcoplasmic reticulum Ca^2+^ release and voltage-dependent Ca^2+^ influx. Together, these effects may reduce smooth muscle contractile sensitivity and favor vasodilation [41,47,48]. The physiological relevance of these endothelial and smooth muscle pathways is particularly important during exercise, when increased shear stress and metabolite accumulation already stimulate endothelial NO release and local vasodilation. Caffeine’s additional modulation of these pathways may therefore alter the magnitude and kinetics of exercise-induced vascular adaptation.

In summary, caffeine’s vascular effects appear to operate across distinct regulatory levels. At the systemic level, adenosine receptor antagonism may increase peripheral resistance, contributing to its pressor effects and influence on cardiac afterload. At the local vascular level, however, enhanced NO signaling, altered cyclic nucleotide metabolism, and changes in smooth muscle Ca^2+^ regulation may partially counterbalance these systemic effects while also modifying exercise-induced vasodilation and perfusion adaptation. The relative contribution of these mechanisms likely depends on vascular bed-specific receptor distribution, the extent to which exercise intensity amplifies metabolic stress signals, and inter-individual differences in adenosine receptor density and reactivity. Consequently, the net hemodynamic and vascular effects of caffeine are not fixed but represent a context-dependent outcome shaped by multiple regulatory factors. Recognizing this complexity is essential for interpreting the heterogeneity observed in the empirical evidence reviewed below.

### 3.3. Metabolic and Substrate-Utilization Pathways

At the metabolic level, pre-exercise caffeine supplementation may influence substrate mobilization and oxidative utilization through multiple interconnected pathways, while also affecting glucose homeostasis during exercise. A central mechanism by which caffeine promotes fat mobilization is its antagonism of adenosine receptors, particularly the A1 subtype, which reduces adenosine-mediated inhibition of sympathetic nervous system activity and increases epinephrine release from the adrenal medulla. After binding to β-adrenergic receptors on adipocyte membranes, epinephrine activates hormone-sensitive lipase (HSL) through the Gs protein–adenylate cyclase–cAMP–protein kinase A (PKA) signaling cascade. This process accelerates triglyceride hydrolysis and increases circulating free fatty acid (FFA) concentrations [49,50]. Importantly, adipocytes also express α_2_-adrenergic receptors, which exert anti-lipolytic effects. Therefore, the magnitude of catecholamine-induced lipolysis depends partly on the relative abundance and activation of β- versus α_2_-adrenergic receptors, representing a potential source of inter-individual variability in lipolytic responses [49]. In addition, in vitro studies have shown a synergistic lipolytic effect between caffeine and epinephrine, suggesting that exercise-induced catecholamine release may amplify caffeine-related signaling within the epinephrine–cAMP–HSL pathway and thereby strengthen its lipolytic effects during exercise compared with rest [51].

Whether increased circulating FFAs translate into altered substrate utilization in skeletal muscle depends partly on the efficiency of fatty acid transport across the sarcolemma. Preclinical evidence suggests that caffeine may promote Ca^2+^ release from the sarcoplasmic reticulum, thereby facilitating the translocation of fatty acid transporters, including CD36, plasma membrane-associated fatty acid-binding protein (FABPpm), fatty acid transport protein 1 (FATP1), and FATP4, to the sarcolemma. This process may enhance the transmembrane uptake of long-chain fatty acids. In CD36 knockout models, caffeine-stimulated muscle palmitate oxidation is markedly attenuated, further supporting the role of CD36-mediated fatty acid transport in caffeine-induced skeletal muscle lipid oxidation [52,53]. After entering the myocyte, fatty acids must be transported into the mitochondrial matrix through a process regulated by carnitine palmitoyltransferase 1 (CPT1), allowing them to enter β-oxidation for energy production [54]. During exercise, competition between free carnitine and glycolysis-derived acetyl-CoA can constrain CPT1 flux. However, greater FFA availability may help maintain acylcarnitine supply within the mitochondrial matrix, thereby creating substrate conditions that favor a higher relative contribution of lipid oxidation [54]. With respect to muscle glycogen utilization, elevated FFA availability may indirectly attenuate the allosteric activation of glycogen phosphorylase by improving muscle energy status during the early stages of exercise, reflected by reduced phosphocreatine depletion and lower ADP/AMP accumulation. Under certain conditions, this may contribute to a muscle glycogen-sparing effect [55,56].

**Figure 2:**
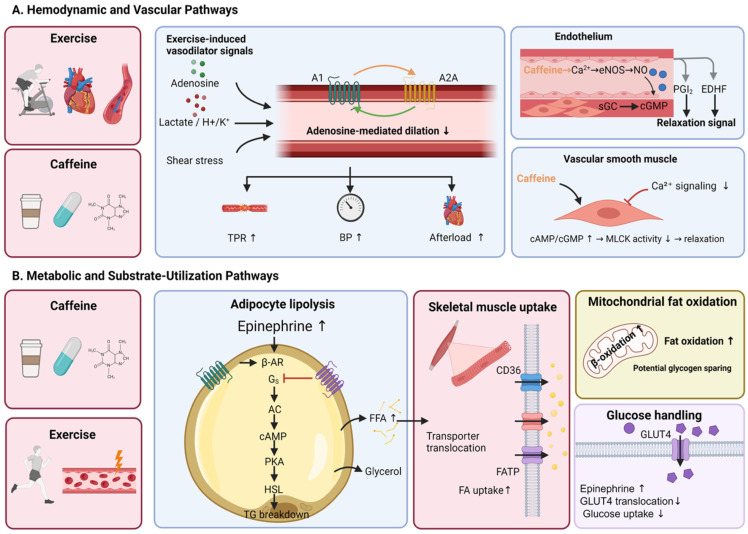
Downstream hemodynamic, vascular, and metabolic pathways through which caffeine reshapes the acute cardiometabolic milieu of exercise. Building on adenosine receptor antagonism (Figure 1), caffeine acts via two parallel downstream axes. (**A**) Hemodynamic–vascular axis: blockade of A1/A2A-mediated functional hyperemia raises total peripheral resistance and arterial pressure, increasing cardiac afterload; endothelial eNOS–NO signaling and vascular smooth muscle relaxation partially counteract this pressor effect. (**B**) Metabolic axis: A1 antagonism elevates sympathoadrenal epinephrine, which activates adipocyte β-AR–cAMP–PKA–HSL signaling to drive lipolysis and free fatty acid (FFA) mobilization, while concurrently suppressing GLUT4 translocation and insulin-dependent skeletal-muscle glucose uptake. The net cardiometabolic outcome reflects context-dependent integration of these opposing pathways. β-AR, β-adrenergic receptor; eNOS, endothelial nitric oxide synthase; GLUT4, glucose transporter type 4; HSL, hormone-sensitive lipase; PKA, protein kinase A; TPR, total peripheral resistance. Arrows indicate the proposed direction of signaling or physiological change; black arrows denote activation or directional flow, red inhibitory lines denote suppression or blockade, and ↑/↓ indicate increases or decreases in the corresponding variables, respectively.

At the level of glucose metabolism, acute caffeine intake may impair insulin-mediated glucose uptake through epinephrine-dependent mechanisms. Epinephrine can inhibit the translocation of skeletal muscle GLUT4 transporters to the sarcolemma, thereby reducing insulin-dependent glucose clearance. This effect does not appear to result primarily from direct disruption of downstream insulin signaling pathways, such as insulin receptor tyrosine kinase (IRTK), PI3K, or PKB/Akt. Rather, it is largely mediated through β-adrenergic receptor signaling, as β-receptor blockade can substantially attenuate caffeine-induced impairments in glucose tolerance. Nevertheless, the independent contribution of adenosine receptor antagonism to skeletal muscle glucose utilization cannot yet be excluded [53,57]. Thus, caffeine’s promotion of lipolysis and suppression of glucose utilization appear to be partly mediated through the epinephrine–cAMP axis (Figure 2). While caffeine may accelerate fat mobilization, it may also impair insulin-dependent glucose regulation during exercise and post-exercise recovery. The extent to which these metabolic effects influence acute cardiometabolic outcomes remains a key issue to be clarified in the subsequent sections of this review.

## 4. Evidence on Acute Cardiometabolic Responses to Pre-Exercise Caffeine

**Figure 3:**
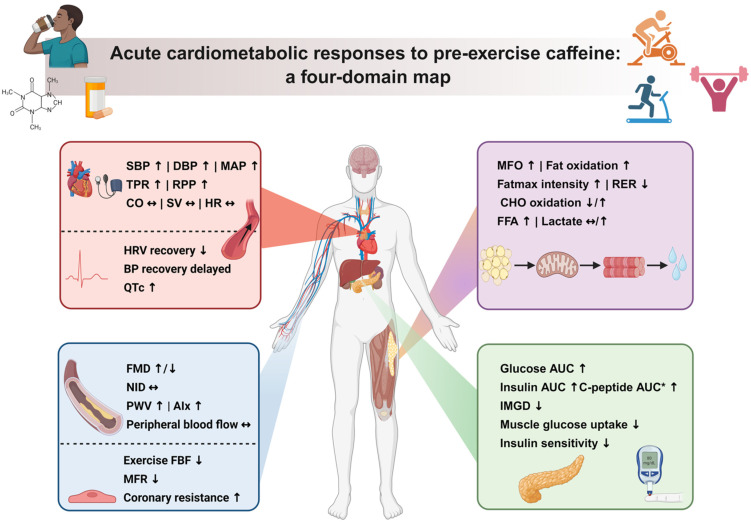
Direction of acute cardiometabolic responses to pre-exercise caffeine across four interconnected physiological domains. Outcomes are coded as upward arrows indicating increases, downward arrows indicating decreases, horizontal bidirectional arrows indicating no apparent change, and combined upward/downward arrows indicating mixed or inconsistent findings across studies. The cardiovascular hemodynamics domain shows that caffeine generally increases blood pressure and total peripheral resistance without clearly altering cardiac output, and may delay post-exercise autonomic and blood pressure recovery. The vascular responses domain indicates short-term increases in arterial stiffness and attenuation of exercise-induced peripheral and coronary perfusion, whereas evidence for flow-mediated dilation remains mixed. The substrate utilization domain shows increases in maximal fat oxidation, Fatmax-corresponding intensity, and free fatty acids, together with reductions in respiratory exchange ratio. The glucose handling domain indicates increased glucose and insulin area under the curve and impaired insulin-mediated skeletal-muscle glucose disposal. Overall, pre-exercise caffeine may magnify circulatory pressor load while concurrently reshaping substrate utilization. AIx, augmentation index; AUC, area under the curve; FFA, free fatty acids; FMD, flow-mediated dilation; HRV, heart rate variability; PWV, pulse wave velocity; RER, respiratory exchange ratio. * limited evidence in the exercise context.

Current evidence from human studies can be organized into three domains: hemodynamic responses, vascular responses, and metabolic responses, with glucose handling considered as a key metabolic outcome (Figure 3).

### 4.1. Hemodynamic Responses

At the hemodynamic level, evidence from acute human studies is relatively consistent: pre-exercise caffeine intake appears to amplify exercise-induced cardiovascular stress primarily by increasing peripheral vascular resistance, rather than by enhancing central pumping function. As early as 1990, Sung et al. [58] reported that, in 34 healthy young men, caffeine ingestion at 3.3 mg/kg significantly increased systolic blood pressure, diastolic blood pressure, and peripheral vascular resistance at rest, without concomitant increases in stroke volume or cardiac output. During subsequent submaximal and maximal exercise, this pressor effect increased in parallel with the blood pressure response induced by exercise itself, leading some participants to exhibit an exaggerated hypertensive response. Later studies in men with mild hypertension further showed that this effect may be more pronounced in individuals susceptible to blood pressure fluctuations, as reflected by greater increases in diastolic blood pressure and rate-pressure product, findings that suggest a potential increase in myocardial oxygen demand [59]. This additive pressor effect is not limited to incremental exercise. During resistance exercise, Souza et al. [60] found that caffeine ingestion at 4 mg/kg increased pre-exercise diastolic and mean arterial pressure and delayed the return of blood pressure to baseline during 9 h of post-exercise ambulatory blood pressure monitoring. Similarly, Ruiz-Moreno et al. [61] reported that 3 mg/kg of caffeine significantly increased systolic, diastolic, and mean arterial pressure during continuous exercise performed at Fatmax intensity. Across the commonly used supplementation range of 3–4 mg/kg, studies have consistently reported directional increases in blood pressure in both aerobic and resistance exercise contexts. Despite differences in participant characteristics, exercise protocols, and measurement time points, current evidence suggests that caffeine’s additive effect on exercise-induced blood pressure responses shows a degree of cross-context reproducibility, with elevated peripheral vascular resistance likely serving as the primary underlying mechanism.

In contrast to blood pressure responses, heart rate and central pumping responses to pre-exercise caffeine intake appear to be more context dependent. At rest or during low-intensity exercise, caffeine is commonly associated with reflexive or modest reductions in heart rate. As exercise intensity increases, however, this difference may diminish and may even reverse during maximal exercise. For example, Bunsawat et al. [62] found that acute ingestion of 400 mg caffeine produced a small increase in maximal heart rate compared with placebo (192 ± 2 vs. 190 ± 2 beats/min). Similarly, Stadheim et al. [63] observed that caffeine increased peak heart rate during incremental exercise to exhaustion. These findings suggest that the heart rate response to caffeine is not unidirectional but depends closely on the exercise phase and testing context. By contrast, several studies measuring stroke volume and cardiac output have reported no significant changes, indicating that the acute hemodynamic effects of caffeine are not primarily mediated by enhanced central pumping function, but are more likely related to changes in peripheral vascular resistance. This vascular-mediated effect may extend beyond the systemic circulation. Using ^15^O-H_2_O PET imaging, Namdar et al. [64,65] showed that as little as 200 mg caffeine can attenuate exercise-induced myocardial blood flow, an effect that was even more pronounced in patients with stable coronary artery disease. These findings suggest that caffeine may simultaneously increase systemic pressure load and reduce local coronary perfusion reserve, potentially exposing susceptible individuals to a dual burden of increased myocardial oxygen demand and limited oxygen supply reserve.

Caffeine may also interfere with post-exercise autonomic recovery and ventricular repolarization. Bunsawat et al. [62] reported that 400 mg caffeine resulted in persistently elevated heart rate, mean arterial pressure, and diastolic blood pressure during the 30 min post-exercise recovery period, accompanied by delayed heart rate variability recovery and mild prolongation of the corrected QT interval. Similarly, Gonzaga et al. [66] observed that caffeine can delay autonomic recovery during specific post-exercise phases, an effect that may be more pronounced in individuals with lower cardiorespiratory fitness. Although a meta-analysis by Porto et al. [67] indicated that no consensus has yet been reached regarding the overall effect of caffeine on post-exercise heart rate variability recovery, existing primary studies suggest that caffeine may delay autonomic recovery under certain doses and exercise conditions.

In summary, the acute hemodynamic effects of pre-exercise caffeine intake are mainly characterized by elevations in blood pressure and peripheral vascular resistance, whereas its influence on central pumping function appears relatively limited. Although heart rate responses, post-exercise autonomic recovery, and coronary microcirculatory responses vary across study contexts, the overall evidence supports a broadly consistent conclusion: caffeine amplifies exercise-induced circulatory stress primarily through vascular-mediated mechanisms.

### 4.2. Vascular Responses

The vascular endothelium is a key site for sensing exercise-induced shear stress and integrating metabolic signals, and it provides an important mechanistic link between changes in blood pressure and peripheral vascular resistance. As discussed above, caffeine may both attenuate endogenous vasodilatory signaling, primarily through adenosine receptor antagonism, and promote vasodilation through cAMP/cGMP- and NO-dependent pathways. This dual potential helps explain the context-dependent nature of its vascular effects.

Evidence on resting endothelial function remains directionally inconsistent. Papamichael et al. [68] reported that, in 17 healthy young adults, regular coffee providing approximately 80 mg of caffeine significantly impaired brachial artery flow-mediated dilation (FMD) within 30–60 min. Notably, nitroglycerin-mediated, endothelium-independent dilation remained largely unchanged, suggesting that the observed effect was mainly related to reduced endothelial NO bioavailability. Using a similar design, Buscemi et al. [69] also observed a transient reduction in FMD, accompanied by changes in plasma antioxidant capacity. By contrast, Shechter et al. [70] reported opposite findings in a larger cohort of healthy controls and patients with stable coronary artery disease: 60 min after ingestion of 200 mg caffeine, FMD increased significantly, accompanied by lower high-sensitivity C-reactive protein levels and higher adiponectin levels. These divergent findings likely reflect not only inter-study variability but also differences in dose, delivery form, baseline endothelial status, and habitual caffeine exposure. In contrast to the mixed evidence for FMD, studies of arterial stiffness and wave reflection provide more consistent directional findings. Vlachopoulos et al. [71] and Mahmud [72] showed that acute caffeine ingestion transiently increases aortic pulse wave velocity (PWV) and augmentation index (AIx), with peak effects typically occurring 30–60 min after ingestion. This time window substantially overlaps with the pre-exercise supplementation period considered in this review. Karatzis et al. [73] further showed in healthy adults that this effect occurs independently of changes in peripheral blood pressure. Notably, the increase appears more pronounced in individuals with hypertension, suggesting that populations with greater baseline vascular stiffness may be more sensitive to caffeine’s acute vasoconstrictive or pressor effects.

However, evidence on vascular responses in the exercise context remains limited. Most studies examining caffeine’s endothelial effects have been conducted under resting conditions, and rigorously controlled randomized trials assessing how caffeine modulates FMD, arterial stiffness, and local perfusion during or after exercise are scarce. From the perspective of exercise-related vascular regulation, limited evidence from coronary microcirculatory imaging suggests that 200 mg caffeine may reduce exercise-induced myocardial blood flow and perfusion reserve, with a concomitant increase in coronary resistance, particularly in patients with stable coronary artery disease. Mechanistically, this finding is consistent with existing evidence on arterial stiffness and raises the possibility that caffeine may attenuate adenosine-mediated functional hyperemia in specific vascular beds during exercise. However, because this evidence is derived from a limited number of studies and specific clinical contexts, it should not be generalized to all exercise settings or populations. In addition, regular exercise training can upregulate eNOS activity and improve FMD through chronic shear stress [74,75]. However, current evidence remains insufficient to determine whether acute caffeine supplementation blunts these training-induced vascular adaptations.

Taken together, pre-exercise caffeine supplementation shows clear directional heterogeneity in vascular responses. Its effects on resting FMD vary, with attenuation or enhancement depending on dose, delivery form, and baseline vascular status, whereas arterial stiffness tends to increase transiently. By contrast, exercise-induced local perfusion responses, as illustrated by coronary microcirculatory findings, may be inhibited. This heterogeneity is unlikely to be merely methodological; rather, it may reflect the hierarchical and context-dependent nature of caffeine’s vascular effects across different physiological settings. Future studies should integrate assessments of limb FMD, arterial stiffness, and local perfusion during and after exercise to clarify the net vascular effect of caffeine on exercise-induced vascular adaptation.

### 4.3. Metabolic Responses

Evidence regarding the modulation of metabolic responses by pre-exercise caffeine intake is complex and multidimensional. This complexity likely reflects caffeine’s simultaneous influence on three interconnected, but not always concordant, pathways: fat mobilization, substrate selection and oxidation, and glucose homeostasis. The observed effects are ultimately shaped by interactions among exercise intensity, caffeine dose, supplementation timing, and individual metabolic characteristics.

When fat oxidation is considered the primary outcome, crossover trials provide the most consistent evidence for moderate-dose caffeine intake, particularly 3 mg/kg, combined with low- to moderate-intensity continuous exercise. Ruiz-Moreno et al. [61] found that, after 1 h of continuous cycling at an individualized Fatmax intensity, 3 mg/kg caffeine significantly increased whole-body fat oxidation while reducing carbohydrate oxidation and the respiratory exchange ratio; perceived exertion was also lower. Ramírez-Maldonado et al. [76] further supported these findings during incremental exercise, showing that the same dose increased both maximal fat oxidation and the exercise intensity at which Fatmax occurred. Notably, this effect was more pronounced during afternoon exercise sessions, suggesting that circadian timing may modulate caffeine’s metabolic effects. In women, Varillas-Delgado et al. [77] reported that both 3 mg/kg and 6 mg/kg caffeine increased maximal fat oxidation, but without a clear dose–response gradient between the two doses. This finding suggests that the 3 mg/kg dose commonly recommended in sports nutrition guidelines may already approach a plateau for fat-oxidation benefits [78]. Consistent with these trial-level findings, several meta-analyses have reported that acute caffeine intake increases fat oxidation during submaximal exercise [18,19]. However, this fat-oxidation-promoting effect is clearly context dependent and may be modified by exercise intensity, substrate availability, and supplementation timing. The study by Yeo et al. [79] provides a representative example: during 2 h of continuous cycling at 64% VO_2_max, co-ingestion of caffeine and exogenous glucose shifted substrate utilization toward carbohydrate oxidation. Specifically, both exogenous and total carbohydrate oxidation increased, whereas fat oxidation decreased; respiratory exchange ratio and blood lactate concentrations also increased. This opposing pattern does not contradict the aforementioned findings. Rather, it suggests that exogenous carbohydrate availability may override caffeine-related lipolytic signaling and shift substrate competition back toward glycolysis. Mechanistically, under these conditions, relatively elevated insulin concentrations may limit net free fatty acid mobilization. In addition, CD36-mediated transmembrane fatty acid transport may be insufficient to match the increased flux of carbohydrate oxidation, thereby attenuating caffeine’s amplifying effects on fat metabolism [52,54].

Another question that has received increasing attention is whether catecholamine-dependent substrate mobilization remains responsive in habitual caffeine users. Van Soeren et al. [11] showed that, even in habitual users consuming more than 700 mg caffeine per day on average, an acute dose of 6 mg/kg caffeine administered after a 0–4-day withdrawal period still significantly increased plasma epinephrine, norepinephrine, and free fatty acid concentrations during exercise, while also prolonging time to exhaustion. Notably, the respiratory exchange ratio and blood glucose concentrations remained unchanged. These findings suggest that the caffeine–catecholamine–lipolysis axis may be relatively insensitive to short-term withdrawal and may retain a robust capacity for acute activation. However, such studies typically involve small samples and predominantly well-trained male endurance athletes. Therefore, extrapolation to women, untrained individuals, or populations with different genetic backgrounds, including CYP1A2 or ADORA2A polymorphisms, requires caution.

More recent experimental evidence further indicates that habitual caffeine use should not be interpreted as a simple binary determinant of acute caffeine responsiveness. Studies stratifying participants by habitual caffeine intake have reported that acute caffeine supplementation can still improve endurance performance in low, moderate, and high-habitual caffeine consumers, suggesting that habitual intake does not necessarily abolish the ergogenic response [80]. However, other experimental work suggests that repeated daily caffeine exposure may reduce the magnitude of some acute responses over time, indicating partial tolerance rather than complete loss of responsiveness [81]. From a cardiometabolic perspective, this distinction is important because tolerance may develop differently across physiological domains. For example, habituation may attenuate some perceptual or performance-related responses, whereas cardiometabolic domains such as catecholamine release, substrate mobilization, blood pressure, glucose–insulin regulation, or recovery-phase autonomic control may not necessarily adapt in parallel. Therefore, habitual caffeine intake should be considered an outcome-specific moderator rather than a uniform suppressor of caffeine’s acute effects. Future studies should quantify habitual caffeine intake, recent abstinence duration, withdrawal symptoms, and circulating caffeine or paraxanthine concentrations when feasible, and should examine whether low and high-habitual consumers differ in both performance and cardiometabolic outcomes during and after exercise.

Overall, the modulation of acute metabolic responses by pre-exercise caffeine intake can be summarized in three key points. First, when moderate-dose caffeine is combined with low- to moderate-intensity aerobic exercise, its fat-oxidation-promoting effect appears directionally consistent and reasonably reproducible. Second, this effect may be substantially attenuated or even reversed when exogenous carbohydrate is co-ingested or when exercise is performed at high intensities dominated by glycolytic metabolism. Third, caffeine’s transient interference with insulin sensitivity and glucose disposal indicates that its metabolic effects should not be evaluated solely in terms of fat oxidation, but rather within a broader framework of altered substrate utilization and glucose regulation.

## 5. Factors Influencing the Response

### 5.1. Caffeine Dose, Timing, and Form of Administration

The commonly recommended caffeine dose for enhancing athletic performance is 3–6 mg/kg [5,82]. Recent reviews further suggest that both lower doses (≤3 mg/kg) and higher doses (6–9 mg/kg) can improve exercise performance, although the magnitude of benefit may differ [4,83]. Spriet noted that smaller caffeine doses do not substantially alter the overall peripheral physiological response to exercise [4,83], suggesting that the performance benefits of low-dose caffeine may arise primarily from central nervous system effects. From a cardiometabolic perspective, however, the peripheral perturbations induced by different caffeine doses are considerably more complex than their central effects. A dose of 3–6 mg/kg is sufficient to acutely increase resting systolic and diastolic blood pressure by approximately 3–15 mmHg and 4–13 mmHg, respectively; this pressor response appears to be driven mainly by peripheral vasoconstriction rather than sympathetic nervous system excitation [84]. At doses of 9 mg/kg or higher, peripheral mechanisms such as phosphodiesterase inhibition, enhanced sarcoplasmic reticulum Ca^2+^ release, and catecholamine surges may become more prominent. These responses are accompanied by increased lipolysis and accelerated free fatty acid release [85], but may also increase the likelihood of arrhythmias, greater aortic stiffness, and adverse gastrointestinal reactions. Thus, the dose–benefit curve for pre-exercise caffeine intake does not appear to progress in parallel across central and peripheral physiological domains.

Supplementation timing is another critical variable. Plasma caffeine concentrations typically peak 30–120 min after ingestion, although substantial inter-individual variability exists because of differences in CYP1A2 enzyme activity, gastric emptying, and whether caffeine is consumed with food [86]. The traditional strategy of ingesting caffeine 60 min before exercise aligns well with the peak plasma concentrations achieved after anhydrous caffeine capsule ingestion. By contrast, caffeine-induced pressor responses typically peak 1–2 h after ingestion and may persist for more than 4 h [84]. This suggests that the cardiovascular exposure window of caffeine is relatively broad, and that the optimal timing of pre-exercise intake may require individualized adjustment. Individuals classified as CYP1A2 slow metabolizers may experience more prolonged vasoconstriction and delayed hypertensive responses. For this subgroup, earlier ingestion, such as 90–120 min before exercise, may help reduce the overlap between peak cardiovascular effects and the exercise bout [87]. Circadian timing should also be considered: early-morning intake may elicit more pronounced hypertensive and cortisol responses, whereas evening intake may be more likely to disrupt sleep architecture and post-exercise autonomic recovery [88,89].

The form of caffeine supplementation also shapes its cardiometabolic profile. Anhydrous capsules and caffeine-containing chewing gum typically produce relatively rapid and pronounced peaks in plasma caffeine concentration. In contrast, when caffeine is consumed within a complex beverage matrix, such as coffee, its pharmacokinetic curve may be flatter. This difference may reflect the modulatory effects of other constituents, including chlorogenic acids, diterpenes, and melanoidins, on caffeine-related vascular responses [90]. Unfiltered coffee, such as espresso or boiled coffee, retains cafestol and kahweol, and long-term consumption may increase low-density lipoprotein cholesterol concentrations. By contrast, paper-filtered coffee largely avoids this adverse lipid-related effect [91]. Energy drinks represent a distinct category of cardiometabolic stimulus because caffeine is combined with other ingredients, such as taurine, guarana, glucuronolactone, and high sugar loads, which may amplify sympathetic activation and QTc prolongation. Case reports also suggest that energy drinks may trigger malignant arrhythmias at caffeine doses that would otherwise be tolerated in the context of standard coffee consumption [92]. In addition, emerging delivery methods, such as oral rinses and nasal sprays, may provide alternative strategies for eliciting central nervous system benefits while minimizing peripheral cardiovascular burden by reducing or bypassing systemic exposure.

It should be noted that these conclusions are largely derived from studies using exercise performance as the primary endpoint. High-quality evidence specifically examining the acute effects of pre-exercise caffeine intake on cardiometabolic parameters remains limited. Future randomized controlled trials should therefore use standardized dose gradients, prespecified ingestion timing, clearly differentiated delivery forms, and cardiometabolic markers as primary endpoints. Such studies would provide a stronger evidence base for developing rational strategies for pre-exercise caffeine use.

### 5.2. Exercise Modality, Intensity, and Protocol

Exercise modality, intensity, and protocol structure collectively determine the physiological context in which caffeine’s effects emerge and are amplified. During low- to moderate-intensity steady-state aerobic exercise, energy supply relies substantially on fatty acid mobilization and oxidation. In this setting, caffeine, through adenosine receptor antagonism, catecholamine release, and enhanced lipolysis, is more likely to increase whole-body fat oxidation. Consistent with this mechanism, evidence indicates that 3 mg·kg^−1^ caffeine can increase fat oxidation, reduce relative carbohydrate oxidation, and lower perceived exertion during exercise performed at Fatmax intensity or during steady-state submaximal cycling and running protocols [20,77]. However, this effect should not be generalized to all aerobic exercise conditions. As exercise intensity increases, exercise duration is prolonged, or exogenous carbohydrate is ingested, glycolytic flux and exogenous glucose oxidation may become more dominant sources of energy. Under these conditions, caffeine’s fat-oxidation-promoting effect may be attenuated or may even shift toward greater carbohydrate utilization [93]. Thus, caffeine should not be regarded simply as a unidirectional driver of fat oxidation; rather, it appears to reshape substrate utilization according to the metabolic demands imposed by a given exercise protocol.

From a cardiovascular perspective, incremental exercise, near-maximal exercise, and high-intensity interval training (HIIT) already impose substantial sympathetic, ventilatory, and blood pressure demands. In these settings, caffeine may act as an additional cardiovascular stressor rather than solely as a metabolic or performance enhancer. For example, Marinho [94] reported that caffeine prolonged time to exhaustion and increased peak ventilation during maximal incremental exercise, whereas effects on peak oxygen uptake, peak heart rate, and blood lactate were inconsistent. Sung et al. [58] similarly showed that caffeine may further increase blood pressure during submaximal to maximal exercise, largely through increased peripheral vascular resistance. Therefore, protocol-specific factors, including interval duration, work-to-rest ratio, repetition number, and recovery measurement timing, should be reported in detail.

Resistance exercise represents another context in which caffeine-related cardiovascular load may be clinically relevant. Because resistance exercise already produces marked pressor responses through breath-holding, mechanical compression, and elevated peripheral resistance, caffeine may further increase pre- or post-exercise blood pressure during high-intensity, multi-set protocols [60]. Caffeine may also delay parasympathetic reactivation, slow blood pressure recovery, and attenuate early post-exercise hypotension [62]. Future studies should therefore report training volume, exercise selection, rest intervals, whether sets are performed to failure, and the observation window used to assess post-exercise recovery.

### 5.3. Participant Characteristics and Responder/Non-Responder Variability

Participant characteristics constitute a key factor in explaining the heterogeneity of responses to pre-exercise caffeine. An identical dose of caffeine does not elicit the same cardiometabolic effects in all individuals; rather, the magnitude of its impact depends on a multifaceted background involving pharmacokinetics, adenosine receptor sensitivity, autonomic nervous system regulation, metabolic flexibility, and tolerance to exercise stress. Consequently, training status, genetic variations, sex, and hormonal status should be regarded as critical moderating variables influencing the interaction between caffeine and exercise.

Within this framework, the concept of responders and non-responders provides a useful, but potentially oversimplified, lens for interpreting inter-individual variability in caffeine responses. In caffeine–exercise research, this concept has most often been used to explain why some individuals show clear ergogenic benefits after caffeine ingestion, whereas others show little benefit or even unfavorable responses under the same experimental conditions. However, responder or non-responder status should not be regarded as a fixed biological label. Rather, it is better understood as an outcome-specific and context-dependent response pattern that depends on the caffeine dose, timing and form of administration, exercise protocol, outcome assessed, baseline physiological status, and within-subject test–retest variability. This distinction is particularly important for acute cardiometabolic outcomes, because ergogenic and cardiometabolic responses may not necessarily align. For example, an individual may experience improved endurance or strength performance after caffeine ingestion while also showing a larger pressor response, increased peripheral vascular resistance, delayed autonomic recovery, altered glucose–insulin regulation, sleep disturbance, or more pronounced adverse symptoms. Therefore, in the context of this review, the key issue is not simply whether an individual is a “responder” to caffeine, but whether caffeine produces a favorable, neutral, or potentially unfavorable cardiometabolic response profile during and after exercise. Such variability may be partly explained by CYP1A2-mediated caffeine metabolism, ADORA2A-related adenosine receptor sensitivity, habitual caffeine intake, training status, sex and hormonal status, baseline blood pressure, metabolic health, and individual tolerance to exercise stress.

Training status may modify caffeine responses, but its direction of influence is unlikely to be uniform. Evidence from trained and untrained athletes suggests that caffeine responsiveness can differ according to training background and performance context [95]. Trained individuals generally exhibit greater neuromuscular efficiency, cardiorespiratory fitness, mitochondrial oxidative capacity, and fatty acid utilization, which may allow them to benefit more readily from caffeine-related central stimulation and substrate mobilization [89,95]. However, training adaptations may also alter adenosine receptor expression or sensitivity, and the effect of a given caffeine dose may therefore vary across training backgrounds [96].

From a cardiometabolic perspective, the interpretation of training status depends strongly on how exercise intensity is prescribed. Under the same absolute workload, trained individuals may show lower heart rate, blood pressure, and metabolic disturbance; under the same relative intensity or near-maximal effort, they may reach higher power outputs and total work, potentially amplifying catecholamine release, lipolysis, ventilation, and blood pressure responses. Therefore, training status should be interpreted together with exercise intensity, workload prescription, total volume, habitual caffeine intake, and the post-exercise recovery window.

Genotype provides further insight into why an identical dose of caffeine elicits divergent responses across individuals. CYP1A2 is the primary enzymatic system responsible for the hepatic metabolism of caffeine; polymorphisms within this enzyme influence both the rate of caffeine clearance and the duration of systemic exposure [86]. Existing gene-stratified studies primarily originate from the field of athletic performance, suggesting that “fast metabolizers” may be more likely to derive performance benefits from caffeine intake; however, such evidence cannot be directly equated with evidence regarding cardiometabolic responses. From a cardiometabolic perspective, a more pertinent concern is that “slow metabolizers” may experience more prolonged caffeine exposure, thereby extending the duration of vasoconstriction, elevated blood pressure, or autonomic nervous system perturbations. Studies have already demonstrated that *CYP1A2* genotype can influence blood pressure responses following caffeine intake, and this effect is further modulated by both physical activity levels and habitual caffeine consumption [97]. Unlike *CYP1A2*, which primarily influences pharmacokinetics, *ADORA2A* polymorphisms operate more at the pharmacodynamic level—specifically, an individual’s sensitivity to the blockade of adenosine A2A receptors. This pathway may influence caffeine-induced alertness, anxiety, sleep disturbance, and subjective feelings of exertion, and may also indirectly affect post-exercise autonomic recovery [98]. Consequently, genotype is not solely relevant to athletic performance; it may also serve as a potential explanatory factor for inter-individual differences in blood pressure responses, vascular regulation, and cardiometabolic profiles during the recovery phase—though direct evidence supporting this remains limited at present.

In current research concerning caffeine and athletic performance, female participants remain underrepresented. Studies have noted that in recent years, women have constituted only 16.3% of all participants in caffeine-performance research—an issue now recognized as a critical priority for future caffeine-related investigations. However, in studies specifically examining the relationship between pre-exercise caffeine intake and acute cardiometabolic responses, independent samples of female participants are even scarcer. This scarcity makes it difficult to directly extrapolate conclusions regarding optimal dosage, timing, and safety—derived primarily from male-centric studies—to the female population. A significant reason for this disparity lies in the inherent complexity of assessing the effects of caffeine in women. Factors such as the use of oral contraceptives, the specific phase of the menstrual cycle, and exogenous hormonal status can all influence *CYP1A2*-mediated caffeine metabolism, thereby altering the duration of systemic exposure to caffeine and its primary metabolites—such as paraxanthine and theophylline. Given that the biological half-life of caffeine may be longer in women than in men, the trajectories of vascular responses, autonomic perturbations, changes in substrate utilization, and residual effects during the recovery phase may differ significantly in women compared to men, even when administered the same dosage [99,100].

Therefore, caution should be exercised when extrapolating findings from studies on men to female athletic populations [101]. Currently, research directly comparing the differences in cardiometabolic responses between men and women following acute pre-exercise caffeine supplementation remains quite limited. Existing evidence regarding sex differences stems primarily from cardiovascular studies conducted under resting or psychological stress conditions, as well as from caffeine studies where exercise performance served as the primary outcome. Although this evidence is not yet sufficient to directly infer the patterns of acute cardiometabolic responses in women within an exercise context, it suggests that men and women may differ in caffeine-induced hemodynamic mechanisms, pharmacokinetic characteristics, and recovery-phase responses. In other words, sex should not be treated merely as a basic demographic variable for sample description, but rather as a critical moderating factor influencing acute cardiometabolic responses to pre-exercise caffeine. Future studies should, at a minimum, systematically report menstrual cycle phase, oral contraceptive use, hormonal status, and time of testing to prevent female-specific pharmacokinetic and cardiometabolic regulatory characteristics from being obscured by overall averaged effects.

Taken together, responder/non-responder variability should be interpreted as a multidimensional and outcome-specific phenomenon rather than a fixed individual trait. Future studies should avoid classifying individuals as responders or non-responders on the basis of a single outcome or a single trial, and should instead report individual-level data, test–retest variability, and responder analyses across both performance and cardiometabolic outcomes.

## 6. Translational Implications and Priorities for Future Research

Integrating mechanistic evidence with findings from human studies raises a practically important question: how should pre-exercise caffeine supplementation be balanced against the need to manage acute cardiometabolic burden? Current evidence suggests that, although the ergogenic effects of caffeine are well established, the acute cardiometabolic context in which these effects occur remains insufficiently characterized. Therefore, practical recommendations should not be based solely on exercise performance outcomes, but should also consider dose, individual cardiometabolic phenotype, and the differential responses elicited by different caffeine delivery forms.

### 6.1. Implications for Exercise Practice and Cardiometabolic Health

First, the ergogenic dose range does not fully overlap with the cardiometabolic safety window; therefore, practice-oriented recommendations should generally favor the lower end of the effective range. Previous studies [4,5,78] suggest that 3–6 mg·kg^−1^ is the most commonly used dose range for improving aerobic, high-intensity, and sport-specific performance, yet doses of approximately 2–3 mg·kg^−1^ may already produce observable ergogenic effects. By contrast, increasing the dose to ≥6 mg·kg^−1^ provides inconsistent additional benefits while increasing the risk of adverse effects. Moreover, acute elevations in blood pressure, impaired flow-mediated dilation, and delayed post-exercise autonomic recovery indicate that higher doses may impose a greater cardiometabolic burden [62]. For athletes with normal baseline cardiometabolic profiles, ingesting approximately 3 mg·kg^−1^ caffeine 45–60 min before exercise may therefore represent a reasonably prudent default strategy. This approach may capture most ergogenic benefits while reducing the risk of moving into a dose range that could exacerbate acute blood pressure responses and the recovery burden. By contrast, some commercially available pre-workout supplements or energy drinks provide 150–300 mg, or even more than 400 mg, of caffeine per serving. Such doses do not necessarily confer additional performance benefits and may instead impose unnecessary cardiometabolic stress.

Second, individual cardiometabolic phenotype should serve as a primary basis, rather than merely a secondary consideration, for decisions regarding caffeine use. Acute caffeine-induced blood pressure responses are notably more pronounced in non-habitual users, individuals with borderline elevated blood pressure, and those with latent hypertension [21]. This has important implications for real-world exercise settings, as many middle-aged recreational exercisers, individuals at metabolic risk, and sedentary office workers rely on coffee, energy drinks, or pre-workout supplements to increase alertness before exercise. For these users, the potential long-term cardiovascular benefits associated with habitual coffee consumption should not be used to offset the immediate risks of acute blood pressure elevation. Moreover, Mendelian randomization studies suggest that the inverse association between coffee intake and cardiovascular risk observed in epidemiological studies may be affected by confounding [102]. Accordingly, individuals with uncontrolled or borderline blood pressure, or those at elevated cardiovascular risk, should prioritize lower doses and avoid initiating caffeine use immediately before high-intensity training or competition. A more prudent strategy would be to introduce caffeine gradually during low- to moderate-intensity training, monitor individual tolerance, and only then consider its use before higher-intensity exercise. Evidence suggests that tolerance to the pressor response may develop over several days to weeks, while the ergogenic effects on exercise performance may be largely preserved [81].

Third, in the absence of sufficient direct comparative evidence, maintaining consistency in the caffeine delivery form has practical value. Anhydrous caffeine, coffee, energy drinks, lozenges, and multi-ingredient pre-workout supplements differ in absorption rate, co-ingested constituents, and pharmacokinetic profile. Although head-to-head trials are currently insufficient to determine whether one delivery form is superior to another in terms of acute cardiometabolic effects, this uncertainty itself suggests that frequent switching between delivery forms may introduce difficult-to-quantify variability in physiological responses. For example, if the same individual alternates among coffee, energy drinks, and caffeine capsules across training sessions, their blood pressure, vascular reactivity, substrate utilization, and recovery responses may not remain consistent. For individuals exercising in hot environments, performing repeated maximal efforts, or presenting with cardiovascular concerns, maintaining a stable caffeine source and dose throughout a training cycle may be a low-cost strategy for minimizing uncontrollable variability. In other words, current evidence does not support treating all caffeine delivery forms as fully interchangeable.

### 6.2. Potential Side Effects and Practical Considerations

Although caffeine is generally well tolerated at commonly used pre-exercise doses, its potential side effects should be considered when interpreting its practical value. The most relevant adverse responses in the context of this review are cardiovascular and autonomic in nature. Acute caffeine intake may increase systolic and diastolic blood pressure, elevate peripheral vascular resistance, delay post-exercise autonomic recovery, and, in susceptible individuals, contribute to palpitations or unfavorable cardiac symptoms [84]. These effects are more likely to be relevant at higher doses, in caffeine-naïve or low habitual users, in individuals with borderline or uncontrolled hypertension, and when caffeine is consumed before high-intensity exercise, resistance exercise, or competition. Therefore, the absence of serious adverse events in young healthy athletes should not be generalized to all exercisers or clinical-risk populations.

Non-cardiovascular side effects should also be considered. Caffeine may cause nervousness, anxiety, tremor, gastrointestinal discomfort, nausea, and sleep disturbance, especially when taken at high doses or close to bedtime [5]. These responses may be amplified in slow caffeine metabolizers or in individuals with higher sensitivity to adenosine receptor blockade. Supplement form is also important: energy drinks and multi-ingredient pre-workout products may contain large caffeine doses together with other stimulatory ingredients, making their cardiometabolic effects less predictable than those of standardized anhydrous caffeine [92]. From a practical perspective, a cautious approach may be to begin with the lower end of the effective dose range, avoid unnecessary dose escalation, avoid late-day ingestion when sleep is a concern, and use caution with high-caffeine energy drinks or multi-ingredient products. For individuals with elevated cardiovascular risk, uncontrolled blood pressure, a history of arrhythmia, or marked caffeine sensitivity, individualized professional guidance may be advisable before using caffeine as a pre-exercise supplement.

### 6.3. Limitations of This Review and the Current Evidence Base

As a narrative review, this article has several inherent methodological limitations that should be acknowledged. Although we used a structured and iterative literature identification process, the search was not designed as a fully systematic or exhaustive search, and no formal protocol registration, risk-of-bias assessment, or standardized grading of study quality was performed. Therefore, the synthesis presented here should be interpreted as a mechanistically informed and integrative overview rather than as a definitive systematic appraisal of the evidence. These limitations may influence the completeness of study identification and the strength with which conclusions can be drawn, particularly given the heterogeneity of caffeine dose, delivery form, exercise modality, participant characteristics, and cardiometabolic outcome measures across the available literature.

The main limitation of the current evidence is not whether caffeine can alter acute cardiometabolic parameters, but rather that key questions remain inadequately addressed: when these changes occur, how long they persist, in which populations they are most evident, and whether they can be reliably predicted.

First, the measurement windows used in many studies are too narrow to capture the full trajectory of acute cardiometabolic responses. Most studies assess outcomes at only a few discrete time points, typically at baseline, immediately after exercise, or during early recovery. Continuous monitoring of blood pressure, heart rate variability, endothelial function, substrate oxidation, and glycemic responses over several hours after caffeine ingestion is uncommon. Given that caffeine has a plasma half-life of approximately 3–7 h and that its metabolites, such as paraxanthine, remain pharmacologically active, later recovery-phase responses may be systematically underestimated. These include autonomic reactivation, return of blood glucose to baseline, and restoration of lipid metabolism. Consequently, conclusions indicating “no significant effect” may reflect an insufficient observation window rather than a true absence of effect.

Second, female participants remain underrepresented, and hormonal status is often poorly controlled. Existing audits indicate that women account for only approximately 16% of participants in studies examining caffeine–exercise interactions [103]. However, oral contraceptives can prolong caffeine half-life by inhibiting CYP1A2 activity [100], and the menstrual cycle phase may influence thermoregulation, vascular tone, and baseline cardiovascular status. Therefore, recommendations on caffeine dose and ingestion timing derived primarily from male cohorts cannot necessarily be extrapolated directly to female athletes or recreationally active women.

Third, habitual caffeine intake is often assessed crudely and without biological validation. Most studies rely on self-report questionnaires and categorize participants as “habitual users” or “non-users” using inconsistent threshold criteria [104]. Yet tolerance to some acute responses may develop over time, while ergogenic or performance-related responses may not change proportionally [81,104]. In addition, psychological expectancy and placebo-related effects may further modify perceived or performance responses to caffeine, complicating the attribution of observed effects to pharmacological caffeine exposure alone [105]. Without objective biomarkers, such as plasma paraxanthine concentrations or urinary caffeine metabolites, it is difficult to interpret heterogeneity in cardiometabolic outcomes across studies. This issue may be even more pronounced in the general population than among athletes, because caffeine sources are more diverse among habitual consumers, including coffee, tea, energy drinks, chocolate, combination analgesics, and other products. This diversity further reduces the accuracy of self-reported intake.

Fourth, although genotype is increasingly recognized as an important effect modifier, it is rarely incorporated into study stratification. Although CYP1A2 and ADORA2A rs5751876 variants have been shown to influence individual responses to caffeine [106,107,108], most acute cardiometabolic trials have not performed genotyping or reported genotype-stratified results. As a result, pooled average effects may obscure clinically meaningful individual differences, particularly in blood pressure responses, vascular regulation, and glucose metabolism. Overall, current evidence remains insufficient to support truly individualized recommendations for pre-exercise caffeine intake.

### 6.4. Future Directions

Given the limitations of the current evidence, future research should move beyond the traditional question of whether caffeine enhances athletic performance. Instead, it should address more translationally relevant questions: in real-world pre-exercise contexts, for which populations, at what doses, through which delivery forms, and under which exercise conditions does caffeine produce predictable acute cardiometabolic benefits or burdens? To this end, the next phase of research should prioritize several areas.

First, dose–response trials stratified by cardiometabolic phenotype are needed. Future studies should move beyond single-dose, placebo-controlled designs and establish clearer dose gradients, such as 0, 3, and 6 mg·kg^−1^, while stratifying participants by blood pressure status, metabolic risk, and habitual caffeine intake. These studies should also incorporate dynamic or ambulatory blood pressure monitoring, continuous glucose monitoring, and post-exercise heart rate variability tracking to capture the full 6–8 h response window after ingestion. Such designs would help determine whether lower doses are sufficient to elicit key ergogenic effects and whether higher doses exacerbate acute cardiometabolic burden in susceptible populations.

Second, the representation of female participants should be improved, and hormonal status should be explicitly incorporated into the study design. Future trials should not only increase the proportion of female participants but also systematically report menstrual cycle phase, oral contraceptive use, and related control strategies. Given that oral contraceptives can prolong caffeine half-life, female participants should not simply be pooled as a subset of the overall sample. Rather, they should be treated as a distinct and important population for understanding variability in acute cardiometabolic responses to caffeine.

Third, habitual caffeine intake should be quantified more precisely. Future studies should avoid simplistic categorization of participants as “users” or “non-users” and instead adopt a more continuous and granular assessment of intake, ideally supported by objective biomarkers such as plasma paraxanthine and urinary caffeine metabolites. Because habitual intake may influence both ergogenic and cardiometabolic responses, and because these responses may not change proportionally, precise control of this variable is essential for interpreting between-study heterogeneity.

Fourth, *CYP1A2 rs762551* and *ADORA2A rs5751876* polymorphisms should be incorporated into standard reporting frameworks. Future studies do not necessarily need to recruit participants in genotype-balanced groups, but relevant genotypes should at least be collected and reported as prespecified covariates. Even when individual studies are underpowered for genotype-stratified analyses, standardized reporting would support subsequent secondary analyses, including individual participant data meta-analyses. Given that several studies suggest that these polymorphisms modulate acute responses to caffeine, failure to account for genotypes may obscure clinically meaningful patterns of individual response.

Fifth, head-to-head comparisons of caffeine delivery forms should be conducted in real-world pre-exercise settings. Future studies could compare anhydrous caffeine, filtered coffee, unfiltered coffee, energy drinks, and multi-ingredient pre-workout supplements while standardizing caffeine dose, for example, at 3 mg·kg^−1^, and using blood pressure, endothelial function, substrate oxidation, heart rate variability, and adverse effects as primary outcomes. Such studies would directly address a common practical question that remains insufficiently supported by empirical evidence: can different caffeine delivery forms be considered equivalent in terms of their acute cardiometabolic effects? Overall, future research should not continue simply to establish whether caffeine is effective, but should instead define clearer boundaries for its individualized use. Only by integrating dose, cardiometabolic phenotype, sex and hormonal status, habitual intake, genotype, and delivery form into the research framework can pre-exercise caffeine supplementation evolve from a generalized sports nutrition recommendation into a more precise tool for balancing performance benefits, cardiometabolic risk, and individualized practice guidance.

## 7. Conclusions

Synthesizing evidence from mechanistic studies and human trials, pre-exercise caffeine should not be regarded simply as a uniformly beneficial ergogenic aid, but rather as a physiological modulator that may reshape the acute cardiometabolic milieu during exercise and recovery. Its effects appear bidirectional and highly context dependent. Moderate doses, including approximately 3 mg·kg^−1^, have been reported to promote lipolysis and fat oxidation during low- to moderate-intensity aerobic exercise in some studies. However, the same dose range may also be associated with higher exercise-induced blood pressure responses, increased peripheral vascular resistance, delayed post-exercise autonomic recovery, and, based on limited evidence, reduced myocardial blood flow or perfusion reserve in susceptible individuals. Thus, performance-related benefits and potentially unfavorable cardiometabolic responses may coexist in some contexts.

Accordingly, the dose range commonly used for ergogenic purposes should not be assumed to be uniformly optimal from a cardiometabolic safety perspective. In practice, a cautious approach may be to begin with the lower end of the effective dose range and avoid unnecessary dose escalation, particularly in individuals with elevated blood pressure, high cardiovascular risk, slow-metabolizer genotypes, or no habitual caffeine use. The central question for future research is no longer simply whether caffeine is effective, but rather: in which populations, at what doses, through which delivery forms, and under which exercise conditions can predictable acute benefits be achieved or additional cardiometabolic load be anticipated? Only by integrating dose–response relationships, cardiometabolic phenotype, sex and hormonal status, habitual intake, and genetic profile into the research framework can pre-exercise caffeine supplementation evolve from generalized sports nutrition advice into a more individualized strategy for balancing performance enhancement and cardiometabolic risk management.

## Data Availability

No new data were generated or analyzed in this review.
